# Molecular detection of *Brucella abortus* in wild and captive felids

**DOI:** 10.1016/j.bjid.2022.102351

**Published:** 2022-04-18

**Authors:** Francielle Cristina Kagueyama, Fernanda Harumi Maruyama, Leticia Camara Pitchenin, Luciano Nakazato, Valéria Dutra

**Affiliations:** Programa de Pós-Graduação em Medicina Veterinária (PPGVET), Faculdade de Medicina Veterinária, Universidade Federal de Mato Grosso (UFMG), Cuiabá, MT, Brazil

**Keywords:** PCR, *Brucella spp*, *Felidae*

## Abstract

**Purpose:**

Brucellosis is a zoonotic disease of great public health importance. In wild animals, *Brucella abortus* is one of the most diagnosed species, mainly in enzootic environments where domestic animals share the same environment. *B. abortus* is common in environments shared by cattle, wild, and domestic animals. This study aimed to detect the presence of *B. abortus* DNA in free-ranging and captivity felids at Mato Grosso State, Brazil.

**Method:**

Polymerase chain reaction, based on the genetic element IS711, was performed in blood samples collected from 23 free-ranging and captive felids. The species represented include *Leopardus colocolo, Leopardus pardalis, Leopardus wiedii, Panthera onca, Puma concolor*, and *Puma yagouaroundi*.

**Results:**

DNA amplification of *B. abortus* was observed in only one captive *P. concolor* (4.34%).

**Conclusion:**

The detection of this pathogen in captive animals using molecular tools demonstrates the importance of monitoring, as it raises concerns about the possibility of transmission between humans and wild and domestic animals, especially in regions of vast biodiversity, such as in the State of Mato Grosso, Brazil.

Zoonotic diseases contribute to 60% of the emerging infectious diseases and out of these 71.8% originate from wildlife. Among pathogens, *Brucella spp*. have great zoonotic potential, with more than 500,000 new cases emerging each year.^1^

In wild animals, *B. abortus* is one of the most diagnosed species, especially in enzootic regions where domestic animals share the same environment.^2^ The presence of infectious pathogens in wild populations contributes to the spread of diseases, decline in species population, and persistence in reservoir hosts.^3^

In recent years, control, eradication, and prevention of brucellosis at the wildlife, livestock, and human interface have been addressed, considering the complex eco-epidemiological aspects of this zoonosis and the importance of wild felids in the maintenance of the functional ecosystem.^4^ This study aimed to detect the presence of *B. abortus* DNA in blood samples from free-ranging and captivity wild felids at Mato Grosso State, Brazil.

Whole blood samples (1 mL) were collected from 23 wild felids between August 2014 and August 2018 in the state of Mato Grosso, Brazil. Of these, 10 from captivity and 13 wilds were rescued by environmental government agencies and admitted for rehabilitation at the Medical Clinic of Wild Animals, Veterinary Hospital, Federal University of Mato Grosso. Animal handling and sample collection were carried out in accordance with the national “Sistema de Autorização e Informação em Biodiversidade” (SISBIO) n° 40617-1 e 42303.

Genomic DNA extraction from the samples was performed using 250 µL of whole blood plus 1 µL of lysis buffer (100 mM NaCl, 25 mM EDTA, 100 mM Tris-HCl pH 8.0, 0.5% SDS, and 0.1 mg proteinase K), which was incubated at 56°C overnight, and subsequently treated with phenol-chloroform.^5^ The DNA was resuspended in 50 μL ultrapure water and stored at -20°C until use.

Polymerase Chain Reaction (PCR) was performed based on the IS711 genetic element from *Brucella abortus* using the following primers: forward 5′-GAC GAA CGG AAT TTT TCC AAT CCC-3′ and reverse 5′-TGC CGA TCA CTT AAG GGC CTT CAT TGC CAG-3′,^6^ which amplified a fragment of 500 bp. Each reaction consisted of 10 ng of DNA, 0.4 pmol of each primer (forward and reverse), 0.2 nM of dNTPs, 3 mM of MgCl_2_, 1 ×  PCR buffer, 1 U of Taq DNA polymerase (Invitrogen), and ultrapure water for obtaining a final volume of 25 μL. The amplification protocol was as follows: initial denaturation for 5 min at 94°C, 35 cycles of denaturation for 15s at 94°C, hybridization for 45s at 60°C, and extension for 30s at 72°C, followed by a final extension cycle at 72°C for 5 min. PCR products were stained with GelRed (Biotium), separated by electrophoresis on 1.5% agarose gel (10 V/cm), and visualized on a transilluminator.

Out of the total sample tested, one (4.34%) captive *P. concolor* was positive for *B. abortus* (Table 1). A likely source of infection in felines raised in captivity is their diet, which is mostly consisted of viscera and fetuse from bovine slaughterhouses.

**Table 1:** Molecular detection by Polymerase Chain Reaction (PCR) of *Brucella abortus* in the blood of wild free-living and captive felids from the state of Mato Grosso, Brazil, during 2014‒2018.

Species (n)	Habitat	Municipality	*Brucella abortus*
*Leopardus colocolo*	Free-living	Várzea Grande	0
*Leopardus pardalis*	Captive	Cuiabá	0
*Leopardus pardalis*	Captive	Cuiabá	0
*Leopardus pardalis*	Captive	Cuiabá	0
*Leopardus pardalis*	Captive	Cuiabá	0
*Leopardus pardalis*	Free-living	Várzea Grande	0
*Leopardus pardalis*	Free-living	Barra do Bugres	0
*Leopardus pardalis*	Free-living	Barra do Bugres	0
*Leopardus pardalis*	Free-living	Várzea Grande	0
*Leopardus wiedii*	Captive	Cuiabá	0
*Panthera onca*	Free-living	Marcelândia	0
*Panthera onca*	Free-living	NF	0
*Panthera onca*	Captive	Cuiabá	0
*Puma concolor*	Free-living	Acorizal	0
*Puma concolor*	Free-living	Tangará da Serra	0
*Puma concolor*	Free-living	Cáceres	0
*Puma concolor*	Free-living	Pontes e Lacerda	0
*Puma concolor*	Captive	Cuiabá	1 (4.34%)
*Puma concolor*	Captive	Cuiabá	0
*Puma concolor*	Captive	Cuiabá	0
*Puma concolor*	Captive	Cuiabá	0
*Puma yagouaroundi*	Free-living	NA	0

NA, Not informed.

Little is known about the prevalence of *Brucella spp*. in wild cats. Reports of *Brucella spp*. in populations of felids detected using PCR are rare. However, *B. abortus* by PCR have been detected in *P. onca* and *L. pardalis*, and *B. canis* in *P. concolor* captive animals.^7^

DNA or antibodies against *Brucella abortus* were detected in some species, such as *Panthera leo* from Tanzania, *P. once, P. concolor* and *L. pardalis* from the Cerrado-biome in Brazil, and *Lynx rufus* from the United States.^7^, ^8^, ^9^, ^10^ Antibodies against *B. canis* were detected in *L. rufus* from the United States, and in *P. concolor* from Brazil.

In the wild, *B. abortus* infections in wild cats are generally associated with predation of infected cattle.^11^ Brucellosis occurs when contact is made between the agent and the respiratory tract, skin lesions, and/or gastrointestinal tract.^12^

Bovine brucellosis in Mato Grosso is associated with beef cattle and is the most frequent infection in animals that share the same habitat with cattle, domestic, and wild animals.^13^ In captivity, a possible source of infection in zoo animals may be associated with the ingestion of contaminated meat and water.^14^

Another risk factor for transmission could be close contact with domestic animals like stray cats, once these animals can access captive animal enclosures and infect them as well as the environment.^2^

The five specimens studied were at extremely high risk of becoming extinct in the wild: *L. colocolo, L. wiedii, P. onca, P. concolor*, and *P. yagouaroundi*. It is noteworthy that *P. concolor* is listed as threatened with extinction in Brazil and are considered vulnerable species.^15^ The impact of wildlife *Brucella* infections on the emergence of brucellosis in animals and humans is difficult to assess, as bacterial transmission is rarely described and poorly understood.^16^

The present study showed that *B. abortus* circulates in wild felines in the state of Mato Grosso. These animals can play an important role in ecological function, as carriers of emerging infectious pathogens and indicators of environmental health, even without the development of clinical disease. The detection of this pathogen in captive animals using molecular tools highlights the importance of monitoring, as it raises concerns about the possibility of transmission between humans and wild and domestic animals, especially in regions of vast biodiversity and ecological interest, such as the state of Mato Grosso.

## Disclaimers

The opinions expressed by authors contributing to this journal do not necessarily reflect the opinions of the Centers for Disease Control and Prevention or the institutions with which the authors are affiliated.

## Conflicts of interest

The authors declare no conflicts of interest.
